# Application of Long-Read Nanopore Sequencing to the Search for Mutations in Hypertrophic Cardiomyopathy

**DOI:** 10.3390/ijms232415845

**Published:** 2022-12-13

**Authors:** Ramil R. Salakhov, Maria V. Golubenko, Nail R. Valiakhmetov, Elena N. Pavlyukova, Aleksei A. Zarubin, Nadezhda P. Babushkina, Aksana N. Kucher, Aleksei A. Sleptcov, Maria S. Nazarenko

**Affiliations:** 1 Research Institute of Medical Genetics, Tomsk National Research Medical Center, Russian Academy of Sciences, 634050 Tomsk, Russia; 2 Cardiology Research Institute, Tomsk National Research Medical Center, Russian Academy of Sciences, 634012 Tomsk, Russia

**Keywords:** hypertrophic cardiomyopathy, long-read sequencing, Oxford Nanopore, sarcomeric protein genes

## Abstract

Increasing evidence suggests that both coding and non-coding regions of sarcomeric protein genes can contribute to hypertrophic cardiomyopathy (HCM). Here, we introduce an experimental workflow (tested on four patients) for complete sequencing of the most common HCM genes (*MYBPC3*, *MYH7*, *TPM1*, *TNNT2,* and *TNNI3*) via long-range PCR, Oxford Nanopore Technology (ONT) sequencing, and bioinformatic analysis. We applied Illumina and Sanger sequencing to validate the results, FastQC, Qualimap, and MultiQC for quality evaluations, MiniMap2 to align data, Clair3 to call and phase variants, and Annovar’s tools and CADD to assess pathogenicity of variants. We could not amplify the region encompassing exons 6–12 of *MYBPC3*. A higher sequencing error rate was observed with ONT (6.86–6.92%) than with Illumina technology (1.14–1.35%), mostly for small indels. Pathogenic variant p.Gln1233Ter and benign polymorphism p.Arg326Gln in *MYBPC3* in a heterozygous state were found in one patient. We demonstrated the ability of ONT to phase single-nucleotide variants, enabling direct haplotype determination for genes *TNNT2* and *TPM1*. These findings highlight the importance of long-range PCR efficiency, as well as lower accuracy of variant calling by ONT than by Illumina technology; these differences should be clarified prior to clinical application of the ONT method.

## 1. Introduction

Hypertrophic cardiomyopathy (HCM) is one of the most common hereditary diseases. The disease can be caused by mutations in many different genes, but more than 50% of identified variants are found in five sarcomeric protein genes: *MYBPC3, MYH7, TPM1, TNNT2,* and *TNNI3* [1,2]. These genes are associated with cardiomyopathy, familial hypertrophic, 4, or CMH4 (OMIM #115197) for *MYBPC3*, CMH1 (OMIM #192600) for *MYH7*, CMH3 (OMIM #115196) for *TPM1*, CMH2 (OMIM #115195) for *TNNT2*, and CMH7 (OMIM #613690) for *TNNI3*. Analysis of these genes is the first-line option in genetic diagnostics of HCM [3].

Currently, the common method for gene sequencing is next-generation sequencing (NGS), but most of the gene panels and exome assays are targeted to coding regions and canonical splice sites, whereas introns and promoter regions are ignored. On the other hand, there is increasing evidence that variants in non-coding regions can contribute to the disease, mostly by altering splicing [4,5,6,7,8,9]. A whole-gene sequence is usually obtained only by whole-genome sequencing, which is more expensive than target gene analysis. Therefore, the development of whole-target-gene sequencing assays would be worthwhile for increasing diagnostic efficiency towards HCM and other hereditary diseases.

The development of single-DNA-molecule long-read sequencing methods opens up new possibilities in the diagnostics of hereditary diseases. Recently, several independent studies were published that describe the application of this method for mutation search in different diseases. For example, Leija-Salazar et al. applied this method to the whole *GBA* gene in Gaucher disease, thereby successfully distinguishing it from a pseudogene [10]. Soufi et al. performed Oxford Nanopore Technology (ONT) sequencing of *LDLR* coding exons in patients with familial hypercholesterolemia [11]. In another paper, hybridisation capture enrichment for the clinical exome (4800 genes) was used with subsequent MinION sequencing, data processing, and variant calling [12]. Targeted long-read RNA sequencing has also been used for evaluation of transcriptional diversity driven by splice-site variations in *MYBPC3* [13] and *DES* genes [14].

Here, we present results of ONT sequencing for five sarcomeric protein genes (*MYBPC3, MYH7, TPM1, TNNT2,* and *TNNI3*) in four patients with HCM in one run, using long-range PCR as an enrichment method. For comparison, we also carried out sequencing of the same PCR products on the Illumina MiSeq platform.

## 2. Results

### 2.1. Long-Range PCR Design

**Figure 1:**
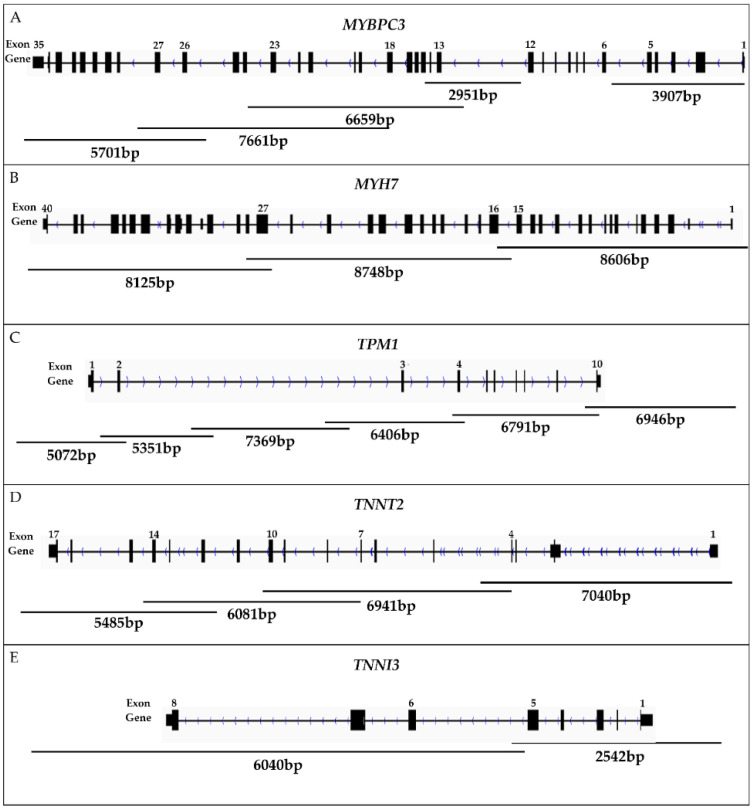
The structure of five sarcomeric protein genes. Primers for long-range PCR employed in this study are illustrated by lines, each annotated with PCR product size (bp). (**A**) The *MYBPC3* gene was amplified in five fragments covering the promoter region and the coding sequences of exons 1–5 and 13–35. (**B**) The *MYH7* gene, including its promoter, was amplified in three fragments. (**C**) *TPM1*, including its promoter, was amplified in six fragments. (**D**) *TNNT2*, including its promoter, was amplified in four fragments. (**E**) The *TNNI3* gene, including its promoter, was amplified in two fragments.

By means of the Primer-BLAST primer design tool on the NCBI website, primer pairs were designed for long PCR products covering the whole sequence of the five sarcomeric protein genes (*MYBPC3, MYH7, TPM1, TNNT2,* and *TNNI3*; Figure 1; Table S1).

After repeated experiments on the adjustment of PCR conditions and testing of different primer pairs, we obtained 20 PCR fragments covering almost complete gene sequences (Figure 1; Table S1). The total length of the target region was 108,329 bp. The total length of amplicons was 124,422 bp.

*MYBPC3* was the most difficult gene for amplification because it took five primer pairs to cover the gene sequence, but long-range PCR was still unsuccessful for the region encompassing exons 6–12 (Figure 1). Further splitting of the region into two PCR products may be required, and, accordingly, this work is in progress.

### 2.2. ONT Sequencing

Sequencing of an equimolar library of the long PCR products (gene fragments) was performed on a MinIon 9.4.1 cell (ONT). A total of 1,143,182 reads were obtained, of which 994,234 (87%) were found to meet quality criteria. The resulting reads constituted 3.54 Gb of sequence data. The average read length was 3560 bases.

**Figure 2:**
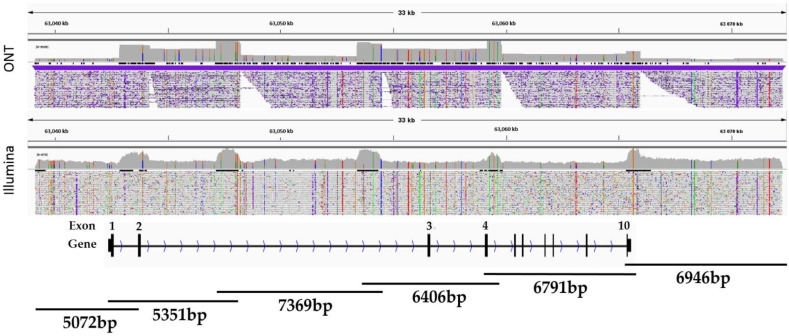
The coverage plot of the long PCR products (gene fragments) for the *TPM1* gene of patient 1.

For all four patients, the mean coverage of the sarcomeric protein genes varied from several hundred to tens of thousands of reads per amplicon (3900× mean coverage for all four HCM patients). Although all amplicons were used in equimolar amounts, we observed a significant coverage imbalance for the target regions of each analysed gene, for example, for *TPM1* (Figure 2).

Moreover, we observed greater variability in coverage between different amplicons than between the same amplicons from different DNA samples. This is probably due to both the length of the fragments and the GC% content or to some problems with sequencing quality for certain gene regions.

Because we were unable to amplify the *MYBPC3* region spanning exons 6 to 12 with a length of ~3500 bp and a >70% GC content, these exons were sequenced by the Sanger method, and we did not find pathogenic variants there. Nevertheless, patient 1 has a benign variant, c.977G>A (rs34580776) in the heterozygous state in exon 12 of the *MYBPC3* gene, leading to the p.Arg326Gln amino acid substitution. This missense variant has been frequently registered in European (Finish) and Slavic populations, including HCM patients (1.5–5%) [15,16].

Then, variant calling was conducted via neural network algorithm Clair3, which, in addition to genotyping, provides information about haplotypes.

### 2.3. Nanopore vs. Illumina Sequencing

To compare different sequencing methods, we sequenced the same PCR products by Illumina’s sequencing technology, thus achieving approximately 300× mean coverage for the target regions in all four HCM patients (Table 1).

A comparison of sequencing statistics between the two methods revealed a higher sequencing error rate for ONT (6.86–6.92%) than for Illumina technology (1.14–1.35%; Table 1). Besides, ONT sequencing detected a much greater number of small deletions and insertions as compared to Illumina sequencing (Table 1). There was a striking difference: Nanopore resulted in 85.5% of reads with at least one insertion (and similarly 85% of reads with at least one deletion) and Illumina resulted in 0.7% and 1.4%, respectively. Even after correction for average read length (which is 3560 bp for ONT and 150 bp for Illumina), there was still a five-fold excess of insertions and a 2.5-fold excess of deletions per read in the ONT data. The high error rate and deletion/insertion rate in the ONT data explain the large number of mismatches as compared to the Illumina data.

Mean mapping quality was the same for both methods (Table 1); differences in read depth are due to a difference in cell capacity and to the presence of other samples in the Illumina run.

Table 2 presents a comparative description of the identified single-nucleotide variants (SNVs) that represent differences from the reference sequence for each gene. In most cases, ONT-identified SNVs matched those identified by Illumina technology. Nonetheless, there were some inconsistencies, and the greatest discrepancy was documented for homopolymer sequences and regions containing tandem repeats.

### 2.4. Pathogenic Variant p.Gln1233Ter in the MYBPC3 Gene

All four patients had hypertrophic cardiomyopathy, according to the results of clinical and ultrasound investigation. In three patients, no likely pathogenic/pathogenic variants were identified in the studied genes.

**Figure 3:**
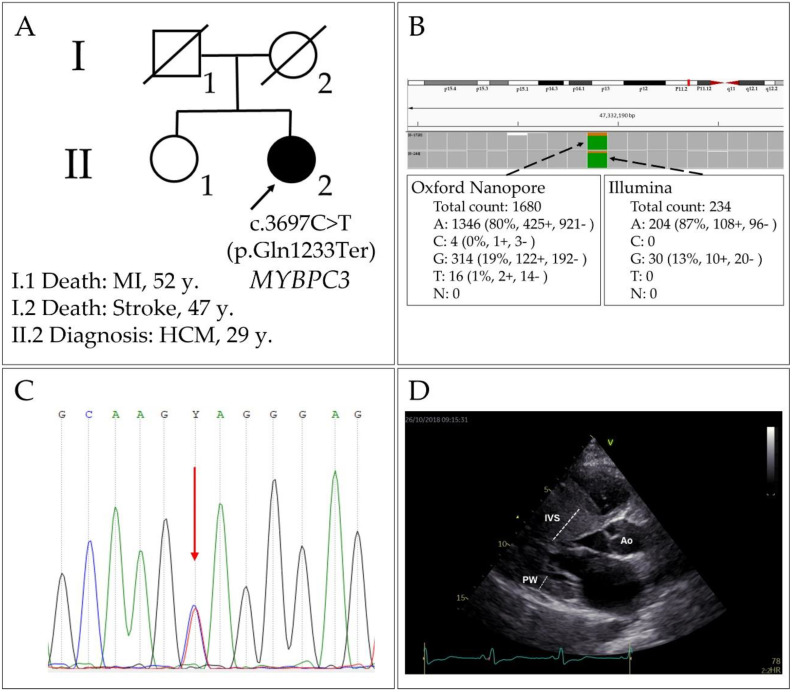
The case of HCM (patient 1) with p.Gln1233Ter (rs397516037) in the *MYBPC3* gene. (**A**) The pedigree of the proband (MI: myocardial infarction); (**B**) molecular genetic testing of the patient by ONT and Illumina methods; (**C**) Sanger sequencing; (**D**) echocardiogram: left ventricle, parasternal long axis position.

We identified a variant in exon 33 of the *MYBPC3* gene [NM_000256.3(*MYBPC3*):c.3697C>T, rs397516037 (p.Gln1233Ter)], which leads to a premature stop codon (CAG→TAG) in patient 1 with HCM (Figure 3A,B) and can be classified as pathogenic, in accordance with the criteria and algorithm presented in the Recommendations for the Interpretation of High-Throughput Sequencing Data [17]. The identified variant was confirmed by Sanger sequencing (Figure 3C). As a result, a truncated protein should be synthesised (lacking 41 amino acid residues). Nevertheless, it is known that, in most cases, mRNAs coding for truncated polypeptides are subject to degradation by the nonsense-mediated decay mechanism, thus implementing the haploinsufficiency mechanism of the mutation effect for *MYBPC3*. For the c.3697C>T (p.Gln1233Ter) variant, a study on septal myectomy samples has shown that only the normal allele transcript (mRNA) is present in the myocardium and that the MYBPC3 protein amount is decreased [18].

The patient harbouring this variant was a 29-year-old female with an obstructive form of HCM and severe asymmetric left-ventricular hypertrophy (interventricular septal thickness 43 mm; Figure 3D); after this diagnosis (in the past), she had undergone surgical correction of the interventricular septum (IVS). This case is characterised by a severe course of the disease: even after the primary surgical intervention, the patient had shown a repeated increase in IVS thickness and high values of the gradient in the left-ventricular outflow tract.

It should be noted that the ONT data analysis indicated that this variant is homozygous with 80% of the variant allele, whereas Illumina data identified it as heterozygous with 87% of the variant allele (Figure 3B). Sanger sequencing verified the heterozygous state of the variant. Accordingly, this case indicates that caution should be exercised when genotypes are identified in the ONT data. The genotype “overcall” might be explained by the fact that, since ONT sequencing quality is not perfect, the genotype calling algorithm assigns a homozygous state by default to the dubious genotypes. In addition, we should note that that such a striking allelic bias in the data (for both methods) could result from the presence of other single-nucleotide polymorphisms (SNPs) in the haplotype with the reference allele; therefore, it can lead either to the primer annealing bias in PCR (partial allelic dropout), if located in the primer-annealing zone [19], or to a context-dependent decrease in sequencing/alignment/base-calling quality.

### 2.5. Haplotype Evaluation

The neural network Clair3 algorithm enables direct phasing of variants. Thus, for the identified variants, haplotypes could be inferred. For patients 1–3, the algorithm was successful, whereas, for patient 4, Clair3 failed to determine the haplotypes for unknown reasons; hence, the genotypes remained unphased for this patient. In most cases, we were able to identify haplotypes spanning the whole genes, except for *MYBPC3*, because a part of this gene was not sequenced with the ONT method.

There were 19 variants (in coding and non-coding regions) that had PHRED > 10, according to CADD results, including two variants in *MYBPC3*, two variants in *MYH7*, one variant in *TNNI3*, six variants in *TNNT2*, and seven variants in *TPM1*. Two variants in *MYBPC3* were variant p.Gln1233Ter (rs397516037) and polymorphism p.Arg326Gln (rs34580776). The two variants in *MYH7* were rs7159367 in intron 29 and rs41285540 in intron 37, and both were found to be situated near branching point sites, indicating a possible effect on splicing. The variant in *TNNI3* (rs11671293) is located in the enhancer region of intron 1.

Given that there were more than two potentially functional significant variants in *TNNT2* and *TPM1*, we analysed haplotype structure in these genes in detail (Table 3). One can see that there is some ‘contrasting’ or clustering of reference and non-reference alleles in different haplotypes, whereas haplotypes containing combined reference and non-reference alleles are less frequent. In *TPM1*, for example, four of six haplotypes/alleles from three patients are formed by reference alleles of rs8026502, rs57645645, rs4075047, and rs111470259 (with one haplotype carrying additional novel variant chr15:63047328A>G and the other carrying additional rs62013181), and the remaining two haplotypes consist of non-reference alleles of all four polymorphisms. These SNPs are located in the region coding for long non-coding RNA TPM1-AS in intron 2. Their population frequencies are similar (9–13% in the non-Finnish European population), suggesting that this linkage pattern can be persistent. Such a linkage disequilibrium could be a consequence of possible selection (in case of intragenic epistasis) or genetic drift, and this issue should be addressed in further studies.

It should be mentioned that the haplotype of the *TNNT2* gene comprising four SNPs (rs1104859, rs3729547, rs4915240 and rs947485) is over 13 kbp. Among them, rs1104859 is associated with electrocardiogram morphology, according to genome-wide association studies [20,21].

## 3. Discussion

In this work, we amplified whole gene sequences for the five most common causative genes of HCM (*MYBPC3, MYH7, TNNT2, TNNI3,* and *TPM1*) by long-range PCR (with 2–6 PCR products per gene) and performed ONT sequencing of these amplicons. To assess accuracy of the sequencing method, we sequenced the same samples (PCR products) on an Illumina MiSeq instrument (with Nextera XT library preparation). Thus, two unusual approaches were employed for target gene sequencing in search of pathogenic variants: long-range PCR for enrichment and the Nanopore long-reads method for the sequencing.

Our approach to the target gene sequencing involves amplification of overlapping long PCR products with maximum length up to 9 kbp, and the amplified region includes a promoter region, exons, introns, and the 3′ untranslated region (3′-UTR). The amplicons were pooled in equimolar quantities and then were barcoded and sequenced by means of ONT, and the data have been processed with rapid bioinformatic analysis. Second- and third-generation sequencing methods have a number of advantages over Sanger sequencing, namely, multiplexing, fast automatic analysis of the whole gene, and low cost per nucleotide. Among the NGS methods, the advantage of long sequencing reads is direct haplotype elicitation.

Despite a reduction in the cost of whole human genome sequencing in recent years, target gene sequencing is still a method of choice for diseases linked with only a small number of causative genes. In such cases, there are many enrichment techniques, including hybridisation capture or multiplex PCR of exonic sequences (as in the AmpliSeq approach). Considering the length of the reads that are obtained by ONT sequencing, we designed PCR primers for long-range PCR (>2000 bp), and the whole PCR product could be sequenced in one read. To ensure full coverage and successful subsequent haplotype evaluation, the amplicons (gene fragments) were designed to overlap by ≥500 bp.

Our PCR approach is similar to the one utilised by Soufi et al. for the *LDLR* gene, although those authors did not aim to sequence the whole gene [11]. Instead, they used long-range PCR to amplify batches of exons, thereby precluding determination of whole-gene haplotypes afterwards [11]. In addition, some deep intronic variants can influence splicing, and, therefore, sequencing the whole gene sequence makes sense, especially for the genes where splice variants are known as disease-causing [5]. On the other hand, amplification of a whole gene takes more effort; consequently, these options should be weighed depending on the disease or gene in question. For instance, a relatively short gene can be amplified in one PCR product, as conducted by Leija-Salazar et al. [10].

The possibility of multiplexing using standard barcodes from a manufacturer (up to 96 indexes) allows one to further reduce the sequencing cost [22]. Unfortunately, we failed to multiplex long-range PCR for several gene regions. For instance, it turned out that the most time-consuming step in this workflow is the obtaining, measuring, and pooling of long-range PCR products for each patient. An effective and cheap method for enrichment of multiplexed long fragments would facilitate the workflow considerably, but such a technique is yet to be developed. Cas9-guided assays are expensive and usually do not permit multiplexing of samples [23]. Adaptation of exome hybridisation panels also works with one sample per sequencing cell [16].

It should be pointed out that, over the past few years, there was a significant improvement of the available bioinformatic and data-processing methods for ONT sequencing. There are several software packages for the processing of raw data, e.g., Nanopolish and Clair3 [24]. Usually, the most efficient protocols are included in ready-made solutions from ONT or are recommended by them. In our work, we used a ready-made software package containing the Clair3 algorithm for evaluating SNVs.

Regarding the applicability of the method of nanopore/single-molecule sequencing to genetic diagnostics, we should mention the difficulties that arise when sequencing regions contain homopolymer sequences. It is known that, when a certain threshold of k-mer signals is exceeded, the received signal is disturbed, thereby limiting the use of this technology for homopolymers with more than four identical bases and lowering sequencing accuracy [25].

Moreover, a high sequencing error rate (together with additional errors introduced by Taq polymerase in long-range PCR), at least in a FLO-MIN106D cell, yields a large number of false positive variants, each of which must be checked by Sanger sequencing. This drawback increases the time and cost of genetic analysis. We hope that further advances in sequencing chemistry will improve the situation. In addition, we faced the problem of amplifying and sequencing regions containing a high proportion of GC pairs, which apparently contribute to reading depth [26]. In particular, we were unable to amplify the region encompassing exons 6–12 of the *MYBPC3* gene, and we are still working on solving this issue.

In addition to the high number of small deletions and insertions reported by ONT sequencing, there are some discrepancies between the methods in the number and spectrum of single nucleotide substitutions (Table 2). It seems likely that the reason for these differences lies in the basecalling algorithms, which are based on the quality of detection of each nucleotide in ONT sequencing. In this regard, there is a possibility of both false-positive and false-negative findings. These circumstances indicate that, for routine genetic diagnostics, the “sequencing by synthesis” method is still preferred, but, in the case of improved sequencing accuracy, nanopore sequencing technology has great prospects for the diagnostics of hereditary diseases, especially in the reconstruction of haplotypes.

**Figure 4:**
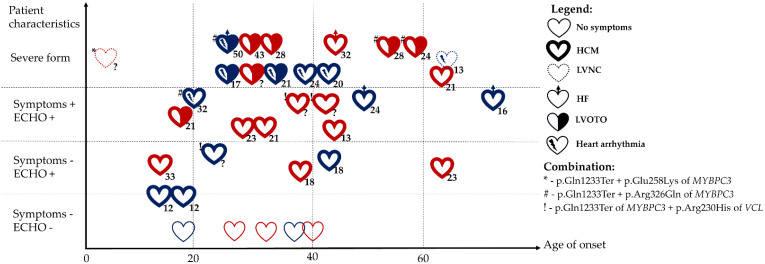
Clinical heterogeneity of HCM in carriers of p.Gln1233Ter of the *MYBPC3* gene [15,27,28,29,30,31,32,33,34,35,36,37,38,39]. Legend. ECHO: echocardiographic signs of myocardium hypertrophy, HCM: hypertrophic cardiomyopathy, LVNC: left ventricular non-compaction, HF: heart failure, LVOTO: left ventricular outflow tract obstruction, ?: no data on left ventricular thickness. Red heart: female; blue heart: male. The numbers outside each heart indicate maximum thickness of the left ventricle (mm).

The nonsense variant p.Gln1233Ter of *MYBPC3*, identified here, has already been described in other reports (Figure 4) [15,27,28,29,30,31,32,33,34,35,36,37,38,39]. HCM-causing MYBPC3 truncation variants, including p.Gln1233Ter, induce a reduction in the amount of cardiac myosin-binding protein C, which enhances maximal myofilament sliding velocities [40].

Several asymptomatic HCM mutation carriers have been reported in the literature (Figure 4). Age of onset of HCM varies among carriers of p.Gln1233Ter of *MYBPC3* from the second decade of life to more than 60 years of age (Figure 4). There is a tendency for earlier onset of the disease in males. The single published case of onset of left ventricular non-compaction in early childhood was a compound heterozygote with p.Gln1233Ter and another *MYBPC3* pathogenic variant leading to p.Glu258Lys substitution [34]. We have previously found that there is accumulation of rare variants in genes associated with arrhythmogenic right ventricular cardiomyopathy in patients with HCM [41]. It must be noted that several patients with symptomatic and severe HCM carrying p.Gln1233Ter of *MYBPC3* also have arrhythmias (Figure 4).

Severe HCM with left ventricular outflow tract obstruction was also detected in a case of compound heterozygosity of p.Gln1233Ter and p.Arg326Gln of *MYBPC3* in one of our patients and in other cases [1,15,31,33,42]. Multiple mutations of *MYBPC3* and other sarcomeric protein genes occurring in HCM-affected families may produce a severer and more complicated clinical phenotype because of a ‘double dose’ effect [1,43,44].

Unfortunately, we could not phase p.Gln1233Ter and p.Arg326Gln in the *MYBPC3* gene by direct haplotyping, owing to the failure of amplification of exons 6–12. It has been suggested that these *MYBPC3* variants are 14,131 bp apart and are present on the same copy of the gene (in *cis*) [15]. The *MYBPC3* p.Arg326Gln variant is classified as benign and has relatively high frequency among Finns and Slavs [15,16]. Nevertheless, the patients’ iPSC-derived cardiomyocytes, with p.Arg326Gln in the *MYBPC3* gene, manifest early HCM signs, such as abnormal calcium handling and an elevated intracellular calcium concentration [42]. It seems that this variant can modify the risk of HCM, and there are additional factors (variants or environmental injury) causing the clinical phenotype of the disease.

It should be underscored that long-read sequencing makes it possible to determine haplotype structure in the studied regions; these data may be helpful in cases where the manifestation of the disease is aggravated by a combination of de novo and inherited variants, as well as in investigation of variation of clinical manifestations in a family. As a result, a cis- or trans-position of pathogenic variants is established, and the haplotypes can be assessed directly [10]. For instance, we were able to determine haplotypes that contain common SNPs, having a possible regulatory function in both *TPM1* and *TNNT2*. Further research is needed to determine the prevalence, structure, and functional effects of these haplotypes in the general population and in patients with heart diseases.

## 4. Materials and Methods

### 4.1. Patients

The study was performed on DNA samples from four patients who obtained a diagnosis of HCM at the Cardiology Research Institute of Tomsk National Research Medical Center (Russia). The patients’ characteristics are presented in Table 4.

### 4.2. DNA Isolation and Amplification

Genomic DNA was isolated from whole blood with the Monarch^®^ HMW DNA Extraction Kit for Cells & Blood (New England BioLabs, Ipswich, MA, USA), followed by assessment of the concentration and purity of the isolated DNA on NanoDrop 8000 (Thermo Fisher Scientific, Waltham, MA, USA). Amplification of fragments of genes *MYBPC3, MYH7, TPM1, TNNT2,* and *TNNI3*, including promoter regions and whole gene sequences, was performed by Long-Range PCR using the Q5 High-Fidelity 2X Master Mix (New England BioLabs, Ipswich, MA, USA) or the BioMaster LR HS-PCR Master Mix (Biolabmix Ltd., Novosibirsk, Russia).

Amplification was performed in a 20 μL reaction mixture consisting of 10 μL of the master mix, 0.5 μL each of forward and reverse primers (Table S1), and 200 ng of genomic DNA. The thermal cycling conditions were as follows: initial denaturation at 95 °C for 1 min, then, 30 cycles of denaturation at 95 °C for 30 s, primer annealing at 58–69 °C for 30 s, and elongation at 68–72 °C (with duration depending on the length of the fragments, assuming a polymerase rate of 1 min/kbp for the calculations), with final elongation for 10 min at 65–72 °C. The amplicons were visualised by 1% agarose gel electrophoresis.

### 4.3. ONT Sequencing

Concentrations of the amplified gene fragments were assayed by means of the BR dsDNA Qubit Kit (Thermo Fisher Scientific, Waltham, MA, USA). After that, the PCR products (100 fmol of each) were pooled (final volume 48 µL) and subjected to library preparation using the Native Barcoding Amplicons Kit (with EXP-NBD104, EXP-NBD114 and SQK-LSK109; Oxford Nanopore Technologies, Oxford, UK), according to the manufacturer’s protocol. The resultant library was loaded into a MinION flow cell (FLO-MIN106D; Oxford Nanopore Technologies, Oxford, UK), and the sequencing was carried out for 48 h.

### 4.4. Processing of ONT Sequencing Data

Base-calling and demultiplexing were performed on the data in the Guppy v.5.0.7 software (Oxford Nanopore Technologies, Oxford, UK). The reads were aligned to reference sequences of the studied genes using MiniMap2 [45].

After alignment of the sequenced data to the reference sequence of the human genome (GRCh38 assembly), a search for genetic variants was performed by two algorithms: Medaka (Oxford Nanopore Technologies, Oxford, UK) and Clair3 (https://github.com/HKU-BAL/Clair3 (accessed on 23 November 2022)). Haplotypes were inferred via phasing by the Clair3 algorithm. Among the identified changes, the variants most likely associated with the disease were selected (missense and nonsense variants described for the first time or registered in populations with a frequency of no more than 0.01%).

To confirm the suspected pathogenicity of the found variants, validation was implemented through PCR combined with Sanger sequencing. For the PCR amplification, primers targeting the variant region were designed (MYBPC3_ex32_F: 5′-CAGCCTTCTGGAAGCTATTGCC-3′ and MYBPC3_ex32_R: 5′-GCATAGTCAGGGACTCTCGTG-3′). The PCR products were sequenced with the BigDye Terminator v3.1 Cycle Sequencing Kit on a 3730 Genetic Analyzer (Thermo Fisher Scientific, Waltham, MA, USA). The results were visualised and interpreted in the UGENE software (UNIPRO, Novosibirsk, Russia) [46].

### 4.5. Library Preparation, Target Sequencing and Bioinformatic Analysis (Illumina)

DNA libraries of the patients were prepared from 200 ng of DNA using the Nextera™ DNA Flex Library Prep, according to the manufacturer’s recommendations (Illumina, San Diego, CA, USA). NGS was performed on the MiSeq Platform (Illumina, San Diego, CA, USA). The data were analysed in accordance with the GATK recommendations [47]. Demultiplexing, alignment of the obtained DNA sequences to the reference genome (GRCh38 assembly), and the search for variants were carried out by means of GATK tools (version 4.2.6.1). Annotation of the identified variants was conducted using Annovar [48]. For the data quality assessment, the following tools were employed: MultiQC v.1.12 [49], FastQC v.0.11.9 [50], and Qualimap v.2.2.1 [51].

### 4.6. Assessment of Pathogenicity of Variants

The identified genetic variants were evaluated in terms of their effect on protein structure and/or function using various annotation tools and databases (Annovar, SIFT, Mutation Tester, MutPred, 1000Genomes, Exome Aggregation Consortium, dbSNP, HGMDB). In addition, potential splice effects for intronic variants were assessed with the help of CADD v.1.6 [52]. Variants with PHRED > 10 were considered for further analysis. The pathogenicity of the identified variants was evaluated based on guidelines for the interpretation of high-throughput sequencing data [17], as well as guidelines for the variant interpretation in cardiomyopathies specifically [53,54].

## 5. Conclusions

We developed an experimental workflow for an analysis of the whole sequence of the five most common HCM genes (*MYBPC3, MYH7, TPM1, TNNT2,* and *TNNI3*). The workflow includes amplification of target regions by means of overlapping gene fragments by long-range PCR, followed by ONT sequencing of these PCR products, variant calling, evaluation, and confirmation. We figured out that there are some issues with PCR efficiency for the *MYBPC3* gene and accuracy of variant calling of sarcomeric protein genes; these problems should be resolved before this method is introduced into routine diagnostics. Nonetheless, there are some advantages as well, including an analysis of non-coding regions and direct haplotype determination. For example, our long-read sequencing-based method was able to also detect common coding and non-coding SNVs with potential functional consequences in genes *TNNT2* and *TPM1* and to phase them into haplotypes.

Using this approach, we analysed these genes in four patients with HCM and found a pathogenic variant in *MYBPC3* (c.3697C>T, rs397516037) leading to a premature stop codon (p.Gln1233Ter). Additionally, another heterozygous benign variant (c.977G>A, rs34580776, p.Arg326Gln) of *MYBPC3* was detected in the same patient. Clinical manifestations of HCM in carriers of *MYBPC3* p.Gln1233Ter vary markedly, ranging from asymptomatic lifelong course to a severe disease phenotype with left ventricular outflow tract obstruction, arrhythmia, heart failure, or even left ventricular non-compaction.

In conclusion, our experience suggests that the third-generation sequencing technology is of considerable interest in terms of the development of target gene panels for the diagnosis of hereditary diseases, owing to its low cost compared to other NGS technologies, easy and fast sample preparation, and the possibility of multiplexing and high coverage of target regions. The ability to detect phased variants, as well as the rapid advances in the data analysis procedures, make this technology promising for diagnostics of hereditary diseases.

## Figures and Tables

**Table 1:** Sequencing and alignment statistics for ONT and Illumina sequencing methods in four samples.

Parameters	ONT/Illumina			
	Patient 1	Patient 2	Patient 3	Patient 4
Mean mapping quality	59.77/59.03	59.73/58.91	59.73/58.89	59.81/58.83
Coverage, mean	3568.7/277.4	3205.6/284.9	3908.2/347.01	4961.2/292.3
General error rate, %	6.9/1.14	6.92/1.23	6.91/1.24	6.86/1.35
Mismatches	19,996,414/333,439	17,905,385/371,635	21,543,356/458,936	27,703,185/417,607
Insertions	3,830,262/3468	3,429,705/3464	4,150,256/3980	5,300,210/3802
Mapped reads with at least one insertion, %	84.93/0.65	83.44/0.54	87.6/0.68	85.61/0.85
Deletions	6,608,331/7444	5,932,133/6932	7,066,882/8140	9,173,356/7264
Mapped reads with at least one deletion, %	85.24/1.39	83.7/1.08	87.89/1.39	85.82/1.64
GC percentage, %	52.12/52.19	51.87/52.1	51.42/51.69	51.28/51.7

**Table 2:** SNV-calling summary for five sarcomeric protein genes of the four patients with HCM.

SNVs	Sarcomeric Protein Genes									
	*MYBPC3*		*MYH7*		*TPM1*		*TNNT2*		*TNNI3*	
	ONT	Illumina	ONT	Illumina	ONT	Illumina	ONT	Illumina	ONT	Illumina
Total	31	35	28	26	99	100	60	58	13	12
Exonic:	3	3	3	3	1	1	2	2	0	0
Synonymous	2	2	2	2	1	1	1	1	0	0
Nonsynonymous	0	0	0	0	0	0	1	1	0	0
Stopgain	1	1	0	0	0	0	0	0	0	0
Splicing sites	0	0	1	1	0	0	0	0	0	0
Intronic	26	30	23	21	78	80	56	54	11	11
3′UTR & downstream	2	2	2	2	20	19	2	2	2	1

**Table 3:** Phased genotypes in genes *TNNT2* and *TPM1*.

No.	Position (GRCh38.p13)	Reference/Alternative Allele	Gene	Location	mRNA Variant	Protein Variant	dbSNP rs#	Genotypes				CADD
								Patient 1	Patient 2	Patient 3	Patient 4	PHRED
1	chr1:201361001	T/C	*TNNT2*	Intron 14/15	-	-	rs10920181	0|1	1|0	0|1	0/0	12.43
2	chr1: 201361301	T/C	*TNNT2*	Exon 13/15	c.A788G	p.R263K	rs3730238	0|1	1|0	0|0	0/0	23.5
3	chr1:201362426	T/G	*TNNT2*	Intron 11/15	-	-	rs1104859	1|1	1|1	1|1	1/1	19.51
4	chr1: 201365254	G/A	*TNNT2*	Exon 9/15	c.C318T	p.I106I	rs3729547	1|1	0|1	1|1	1/1	10.80
5	chr1:201371368	G/A	*TNNT2*	Intron 3/15	-	-	rs4915240	1|1	0|1	0|1	0/1	11.79
6	chr1:201376224	A/G	*TNNT2*	Intron 2/16 (1/15)	-	-	rs947485	1|1	1|1	1|1	0/1	10.25
7	chr15:63042489	G/C	*TPM1*	upstream 258 b.p.	-	-	rs35829897	0|0	0|0	0|0	0/1	18.82
8	chr15:63043900	C/G	*TPM1*	Intron 1/8	-	-	rs62013181	0|1	0|0	0|0	0/0	18.96
9	chr15:63046558	T/C	*TPM1/TPM1-AS*	Intron 2/8	-	-	rs8026502	1|0	0|0	1|0	0/1	11.46
10	chr15:63047328	A/G	*TPM1/TPM1-AS*	Intron 2/8	-	-	-	0|0	0|0	0|1	0/0	15.04
11	chr15:63047379	G/A	*TPM1/TPM1-AS*	Intron 2/8	-	-	rs57645645	1|0	0|0	1|0	0/1	15.48
12	chr15:63048408	T/C	*TPM1/TPM1-AS*	Intron 2/8	-	-	rs4075047	1|0	0|0	1|0	0/1	13.03
13	chr15:63048506	C/T	*TPM1/TPM1-AS*	Intron 2/8	-	-	rs111470259	1|0	0|0	1|0	0/1	14.44

**Table 4:** Clinical data on the HCM patients.

Patient Characteristics	Patient 1	Patient 2	Patient 3	Patient 4
Age (years old)/sex (f-female; m-male)	29/f	54/m	63/m	35/m
Interventricular septal thickness, mm	43	19	12	35
Left ventricular posterior wall thickness, mm	12	18	13	23
Left ventricular outflow tract gradient rest/Valsalva manoeuvre, mmHg	9.6/5.51	66.27/165	21.39/51.47	11.71/10.51
Left ventricular myocardial mass, g	213.5	184	140	239
Left ventricular ejection fraction, %	73	61	79	71
Septal reduction therapy	yes	yes	yes	no
Symptoms	Angina, dyspnoea, palpitations	Angina, dyspnoea	Angina, dyspnoea, palpitations	Dyspnoea

## Data Availability

Raw data are available from the corresponding author upon request.

## Supplementary Materials

The following supporting information can be downloaded at: https://www.mdpi.com/article/10.3390/ijms232415845/s1.
